# Prognostic factors for the severity of SARS-CoV-2 infection

**DOI:** 10.1515/almed-2021-0017

**Published:** 2021-03-03

**Authors:** Ricardo Rubio-Sánchez, Esperanza Lepe-Balsalobre, María del Mar Viloria-Peñas

**Affiliations:** Clinical Laboratory, Virgen de Valme University Hospital, South Health District of Seville, Seville, Spain

**Keywords:** coronavirus, COVID-19, infection, laboratory, SARS-CoV-2, severity

## Abstract

**Objectives:**

Severe acute respiratory syndrome coronavirus (SARS-CoV-2) is a novel coronavirus that causes COVID-19. This disease is associated with leukocytosis with lymphopenia, neutrophilia, and elevated levels of d-dimer, and C-reactive protein, ferritin, procalcitonin, and lactate dehydrogenase. The aim of this study was to describe the clinical and analytical characteristics of hospitalized patients with SARS-CoV-2 infection and to identify prognostic factors of disease progression.

**Methods:**

Patients were categorized into two groups based on COVID-19 severity. Study variables included demographic data, medical history, length of hospital stay, course of pneumonia, drug therapy, and analytical parameters. A descriptive and multivariate analysis was performed to identify prognostic factors for disease severity.

**Results:**

The study population included 197 patients, of whom 127 had mild disease and 70 had severe COVID-19. Statistically significant differences were observed in most analytical parameters. The parameters included in the multivariate analysis were advanced age and elevated levels of leukocytes, CRP, GGT, and PCT at admission as prognostic factors for disease severity.

**Conclusions:**

The prognostic factors for the severity of SARS-CoV-2 infection identified in this study (age, leukocytes, CRP, GGT, and PCT) will help predict the course of the disease at an early stage.

## Introduction

SARS-CoV-2 is a novel coronavirus of the *Coronaviridae* family that was first identified in humans with symptoms of viral pneumonia in Wuhan, China, in January 2020. This virus belongs to the betacoronavirus gender, which also includes severe acute respiratory syndrome coronavirus (SARS-CoV) and Middle East respiratory syndrome-related coronavirus (MERS-CoV) [1]. On March 11, 2020, the World Health Organization (WHO) declared it a global pandemic [1], [2].

The disease caused by SARS-CoV-2 is known as COVID-19, characterized by the presence of fever, cough, and dyspnea. However, patients with COVID-19 may remain asymptomatic, and 20% develop severe symptoms such as interstitial pneumonia and severe acute respiratory syndrome. In addition, the disease may progress into respiratory failure with multiorgan failure and death, especially in elderly patients or patients with underlying comorbidities. Therefore, the identification of diagnostic and prognostic biomarkers of SARS-CoV-2 infection is crucial [3], [4].

SARS-CoV-2 infection activates the innate immune system, triggering an overreaction that leads to deregulation of the cytokine cascade. These events cause damage to the microvascular system, activate the coagulation system and inhibit fibrinolysis. Patients with COVID-19 exhibit elevated levels of proinflammatory cytokines associated with T-cell decline, pulmonary inflammation, and large pulmonary damage [5], [6]. A variety of studies demonstrate elevated levels of acute phase reactants, which leads to liver dysfunction, inducing disseminated intravascular coagulation in patients with SARS-CoV-2 infection [6], [7].

As to analytical parameters, leukocytosis with lymphopenia, which is probably caused by translocation in peripheral blood lymphocytes to the lungs, and neutrophilia were frequently observed in COVID-19 patients. Additionally, patients with SARS-CoV-2 infection showed elevated levels of d-dimer, erythrocyte sedimentation rate, C-reactive protein, ferritin, procalcitonin, transaminases, bilirubin, creatinine, and lactate dehydrogenase. These patients also exhibited reduced platelet and hemoglobin count, and prolongation of prothrombin time [2], [5], [6], [7], [8].

The aim of this study was to describe the clinical and analytical characteristics of hospitalized patients with SARS-CoV-2 infection in the province of Seville, Spain, and to identify prognostic factors for disease progression.

## Materials and methods

### Study design and population

This is a descriptive, retrospective, and cross-sectional study conducted at Virgen de Valme University Hospital (Seville, Spain) involving patients with SARS-CoV-2 infection admitted to the hospital between March 14 and June 5, 2020. This study was carried out in accordance with the principles of the Declaration of Helsinki and was approved by the Ethics Committee of Sevilla Sur (No. 1447-N-20).

Inclusion criteria were: patients of all ages with SARS-CoV-2 infection confirmed by reverse transcription polymerase chain reaction (RT-PCR) who required hospitalization. Patients with suspected infection and negative RT-PCR results were excluded.

The patients included in the study were classified into two groups based on infection severity, as patients with mild disease (ward admission) and patients with severe disease (ICU admission) and/or death.

### Data collection

Clinical data, nursing records, and laboratory results were collected from the electronic medical records of patients. Two independent investigators reviewed all the data collected. The variables studied were:

– Demographic data: age and sex.
– Medical history: arterial hypertension, dyslipidemia, cardiovascular disease, diabetes mellitus, chronic renal insufficiency, cancer, vascular disease, pulmonary disease, thyroid disease, liver disease, and anemia.
– Length of hospital stay (days), length of ICU stay, and course of pneumonia.
– Drug therapy: hydroxychloroquine, lopinavir/ritonavir, corticosteroids, azithromycin, tocilizumab, interferon beta, cyclosporine, and anakinra.
– Analytical parameters: leukocytes, lymphocytes, neutrophils, hemoglobin, platelets, erythrocyte sedimentation rate (ESR), prothrombin time (PT), activated partial thromboplastin time (APTT), d-dimer, creatinine, total bilirubin, alanine aminotransferase (ALT), aspartate aminotransferase (AST), γ-glutamyltransferase (GGT), lactate dehydrogenase (LDH), C-reactive protein (CRP), procalcitonin (PCT), and ferritin.

### Laboratory tests

Blood samples were drawn and a nasopharyngeal specimen was obtained from all patients included in the study. Blood was drawn by venous puncture and stored in three different tubes: one with EDTA for blood cell counting and ESR on the Sysmex XN-2000 analyzer (Sysmex, Kobe, Japan); another tube with sodium citrate for the study of PT, APTT, and d-dimer on the Sysmex CS-5100 analyzer (Sysmex, Kobe, Japan) by immunoassay; and another tube with lithium heparin for the analysis of biochemistry in plasma on the Hitachi Cobas c702 modular analyzer (Roche Diagnostics, Rotkreuz, Switzerland): creatinine, total bilirubin, ALT, AST, GGT, and LDH by photometry, while CRP, PCT, and ferritin were assayed by immunoturbidimetry.

The nasopharyngeal exudate was obtained using two throat swabs impregnated with Copan eNATM for RT-PCR for SARS-CoV-2. Viral ARN was extracted from samples using the pure compact RNA isolation kit (Roche Diagnostics, Rotkreuz, Switzerland). A quantitative RT-PCR was performed using primers and probes targeted to SARS-CoV-2 *ORF*1ab and *N* genes. In compliance with the guidelines of different centers for disease control and prevention, a specific commercially-available kit was used for the detection of SARS-CoV-2 (VIASURE CerTest BIOTEC). Specific primers and a fluorescence probe were employed. Samples were positive if an amplification signal was detected on the detection system and internal control. Samples were negative in the absence of any amplification signal on the detection system and a positive result on internal control. The assay was repeated in the presence of a negative control amplification signal or in the absence of signal in the positive control.

### Statistical analysis

Data analysis was performed using the SPSS Statistics 25.0 software package (IBM, Chicago, USA). Categorical variables are described as absolute values (n) and percentages (%). Comparison of these variables between groups was performed by Pearson’s Chi-square. Continuous variables are expressed as mean values and interquartile ranges (IQR). Comparisons were performed using Student’s t*-*test for variables with normal distribution and Mann-Whitney U test for variables with abnormal distribution. Normality of variables was determined by the Kolmogorov–Smirnov test.

Univariate and multivariate analyses of prognostic factors were performed by logistic regression. The multivariate analysis was based on the backward stepwise method (conditional), including in the initial step all the variables that showed a univariate association with a p-value<0.05. A p-value>0.10 was established for sequential exclusion of variables from the multivariate model. The risk of disease progression assigned to each variable is expressed as odds ratio (OR), next to the 95% confidence interval (CI).

Cut-off points for logistic regression were established on the basis of the different values close to the cut-off point with the highest Youden index. To such purpose, variables were dichotomized using round cut-off points. The cut-off points used in logistic regression were selected based on the cut-offs with the highest likelihood on the statistical model.

The area under the curve (AUC), sensitivity, and specificity of the parameters included in logistic regression were analyzed by the receiver operating characteristic curve (ROC). Level of significance was set at a p value<0.05.

## Results

The sample was composed of 197 patients with ages ranging from 10 to 98 years old (median=72), of whom 97 were male and 100 were female. Sex-based distribution in the two groups was similar, whereas there were statistically significant differences in age, being the median age in the group of patients with mild disease 67 years old vs. 78 in the group of critical patients.

In total, 64.47% (127/197) had mild disease vs. 35.53% (70/197) who had severe disease, of whom 49 died and 21 were admitted to the ICU with endotracheal intubation. Out of the patients who stayed in the ICU, 12 were discharged, whereas nine died. In general, 29.44% (58/197) of patients died. The most frequent cause of mortality was cardiac arrest or acute respiratory distress (ARDS).

Arterial hypertension was the most common comorbidity (58.4%), followed by abnormal lipid profile (35.0%), cardiovascular disease (28.9%), and diabetes mellitus (27.4%). Statistically significant differences were observed in chronic renal insufficiency, cancer, vascular disease, and liver disease, being the incidence of these comorbidities higher in the group of critical patients. Table 1 contains the demographic data and medical history of patients.

**Table 1:** Demographic data and clinical characteristics of patients.

	Total n=197	Mild n=127	Critical n=70	p-Value
Age, years, median (IQR)	72 (59–84)	67 (57–80)	78 (68–86)	0.001
Sex (man/woman)	97/100	58/69	39/31	0.177
Comorbidities, n (%)				
Arterial hypertension	115 (58.4)	70 (55.1)	45 (64.3)	0.272
Dyslipidemia	69 (35.0)	39 (30.7)	30 (42.9)	0.087
Cardiovascular disease	57 (28.9)	32 (25.2)	25 (35.7)	0.237
Diabetes mellitus	54 (27.4)	31 (24.4)	23 (32.9)	0.203
Chronic renal insufficiency	27 (13.7)	12 (9.4)	15 (21.4)	0.019
Cancer	22 (11.2)	10 (7.9)	12 (17.1)	0.048
Vascular disease	19 (9.6)	5 (3.9)	14 (20.0)	0.001
Pulmonary disease	18 (9.2)	11 (8.7)	7 (10.0)	0.755
Thyroid disease	18 (9.2)	9 (7.1)	9 (12.9)	0.179
Liver disease	18 (9.2)	7 (5.5)	11 (15.7)	0.017
Anemia	13 (6.6)	8 (6.3)	5 (7.1)	0.819

IQR, interquartile ranges.

The mean length of hospital stay was eight days, increasing to 27 days for ICU patients. The median length of stay in the ICU was 20 days. A total of 83.8% of patients developed bilateral community-acquired pneumonia (CAP) secondary to SARS-CoV-2 infection. CURB-65 is an index of severity used for CAP based on patient’s age, confusion, respiratory rate, ureic nitrogen concentration, and systolic and diastolic blood pressure. Most patients with pneumonia had a CURB-65 of two points, although this percentage was higher in critical patients (52.5%), as compared to in patients with mild disease (34.9%) (Table 2).

**Table 2:** Progression to pneumonia and treatment administered.

	Total n=197	Mild n=127	Critical n=70
Pneumonia, n (%)	165 (83.8)	106 (83.5)	59 (84.3)
CURB-65=0	31 (18.8)	26 (24.6)	5 (8.5)
CURB-65=1	44 (26.7)	34 (32.0)	10 (17.0)
CURB-65=2	68 (41.2)	37 (34.9)	31 (52.5)
CURB-65=3	22 (13.3)	9 (8.5)	13 (22.0)
Treatment, n (%)			
Hydroxychloroquine	161 (81.7)	110 (86.6)	51 (72.9)
Lopinavir/Ritonavir	94 (47.7)	62 (48.8)	32 (45.7)
Corticosteroids	58 (29.4)	24 (18.9)	34 (48.6)
Azithromycin	27 (13.7)	7 (5.5)	20 (28.6)
Tocilizumab	12 (6.1)	0 (0.0)	12 (17.1)
Interferon β	10 (5.1)	5 (3.9)	5 (7.1)
Ciclosporin	4 (2.0)	3 (2.4)	1 (1.4)
Anakinra	3 (1.5)	1 (0.8)	2 (2.9)

During admission, no data from controlled clinical trials were available supporting the use of any specific treatment for SARS-CoV-2. Therefore, the therapies administered were under study and were used as established in local protocols. The drug most frequently used in COVID-19 patients during admission was hydroxychloroquine (81.7%), followed by the combination of lopinavir and ritonavir (47.7%) and the group of corticosteroids (29.4%). Azithromycin and tocilizumab were reserved for patients with a poor prognosis who were unresponsive to baseline therapy (Table 2).

Table 3 shows analytical results for the two groups. There were statistically significant differences between the two groups in hematological (leukocytes, neutrophils, hemoglobin, ESR), hemostatic (PT, APTT, D-dimer), and biochemical (creatinine, total bilirubin, GGT, LDH, CRP, PCT, ferritin) parameters. There were no statistically significant differences between the two groups in the following analytical parameters: lymphocytes, platelets, and transaminases (ALT and AST).

**Table 3:** Analytical results at admission.

	Total n=197	Mild n=127	Critical n=70	p-Value
Analytical parameters (median, IQR)				
Leukocytes, ×10^9^/L	6.76 (4.78–9.10)	6.15 (4.30–7.84)	8.74 (6.14–11.93)	<0.001
Lymphocytes, ×10^9^/L	0.96 (0.75–1.34)	1.01 (0.77–1.31)	0.91 (0.63–1.44)	0.320
Neutrophils, ×10^9^/L	5.35 (3.15–7.48)	4.49 (2.79–6.25)	7.23 (4.34–10.67)	<0.001
Hemoglobin, g/L	131 (119–144)	133 (121–146)	126 (115–137)	0.004
Platelets, ×10^9^/L	203 (158–271)	201 (157–269)	209 (160–275)	0.496
ESR, mm/h	47 (16–70)	36 (15–64)	56 (19–84)	0.024
PT, s	12.0 (11.4–13.0)	11.9 (11.4–12.6)	12.6 (11.8–13.7)	0.002
APTT, s	32.1 (28.8–37.0)	31.4 (28.5–36.0)	33.4 (29.8–38.2)	0.047
d-dimer, mg/L	0.91 (0.54–1.92)	0.80 (0.47–1.38)	1.54 (0.71–3.42)	<0.001
Creatinine, µmol/L	83.1 (63.6–115.8)	76.9 (63.6–104.3)	107.9 (72.5–136.1)	0.001
Total bilirubin, µmol/dL	6.8 (5.1–10.3)	6.8 (5.1–10.3)	7.7 (5.1–12.5)	0.009
ALT, U/L	23.0 (14.0–34.4)	23.0 (14.1–34.0)	23.0 (13.6–37.0)	0.968
AST, U/L	33 (23–50)	31 (23–46)	36 (23–54)	0.331
GGT, U/L	39 (23–86)	31 (21–67)	54 (29–125)	0.002
LDH, U/L	294 (238–411)	288 (234–365)	322 (242–538)	0.023
CRP, mg/L	69.3 (24.4–140.8)	51.3 (17.9–120.5)	117.6 (50.2–247.8)	<0.001
PCT, ng/mL	0.12 (0.08–0.29)	0.09 (0.06–0.14)	0.31 (0.16–0.73)	<0.001
Ferritin, µg/L	507 (244–1,052)	462 (224–781)	677 (276–1,335)	0.012

ESR, erythrocyte sedimentation rate; PT, prothrombin time; APTT, activated partial thromboplastin time; ALT, alanine aminotransferase; AST, aspartate aminotransferase; GGT, γ-glutamyltransferase; LDH, lactate dehydrogenase; CRP, C-reactive protein; PCT, procalcitonin.

**Table 4:** Logistic regression of prognostic factors of severity.

	Univariate analysis			Multivariate analysis		
	β	OR 95%CI	p-Value	β	OR 95%CI	p-Value
Age, years						
<70		1 (ref)			1 (ref)	
≥70	1.129	3.094 (1.645–5.819)	<0.001	0.823	2.277 (1.022–5.076)	0.044
Leukocytes, ×10^9^/L						
<9.5		1 (ref)			1 (ref)	
≥9.5	2.241	9.405 (4.328–20.441)	<0.001	1.884	6.577 (2.605–16.608)	<0.001
CRP, mg/L						
<90		1 (ref)			1 (ref)	
≥90	1.528	4.611 (2.467–8.616)	<0.001	1.022	2.779 (1.299–5.949)	0.008
GGT, U/L						
<30		1 (ref)			1 (ref)	
≥30	1.027	2.792 (1.461–5.337)	0.002	0.892	2.440 (1.062–5.604)	0.036
PCT, ng/mL						
<0.5		1 (ref)			1 (ref)	
≥0.5	3.080	21.750 (7.215–65.571)	<0.001	2.450	11.590 (3.439–39.054)	<0.001

OR, odds ratio; CI, confidence interval; CRP, C-reactive protein; GGT, γ-glutamyltransferase; PCT, procalcitonin.

In the multivariate analysis, the parameters at admission most strongly associated with severity of SARS-CoV-2 infection were age≥70 years (OR=2,277; IC 95% 1,022–5,076), leukocytes≥9.5 × 10^9^/L (OR=6,577; IC 95% 2,605–16,608), CRP≥90 mg/L (OR=2,779; 95%CI 1,299–5,949), GGT≥30 U/L (OR=2,440; 95%CI 1,062–5,604), and PCT≥0.5 ng/mL (OR=11,590; 95%CI 3,439–39,054). On the logistic regression model, PCT showed the highest coefficient, followed by leukocyte count.

The univariate analysis also identified PCT as the parameter most strongly associated with severity of SARS-CoV-2 infection (OR=21.750; 95%CI 7.215–65.571). Table 4 shows the results of logistic regression.

**Figure 1:**
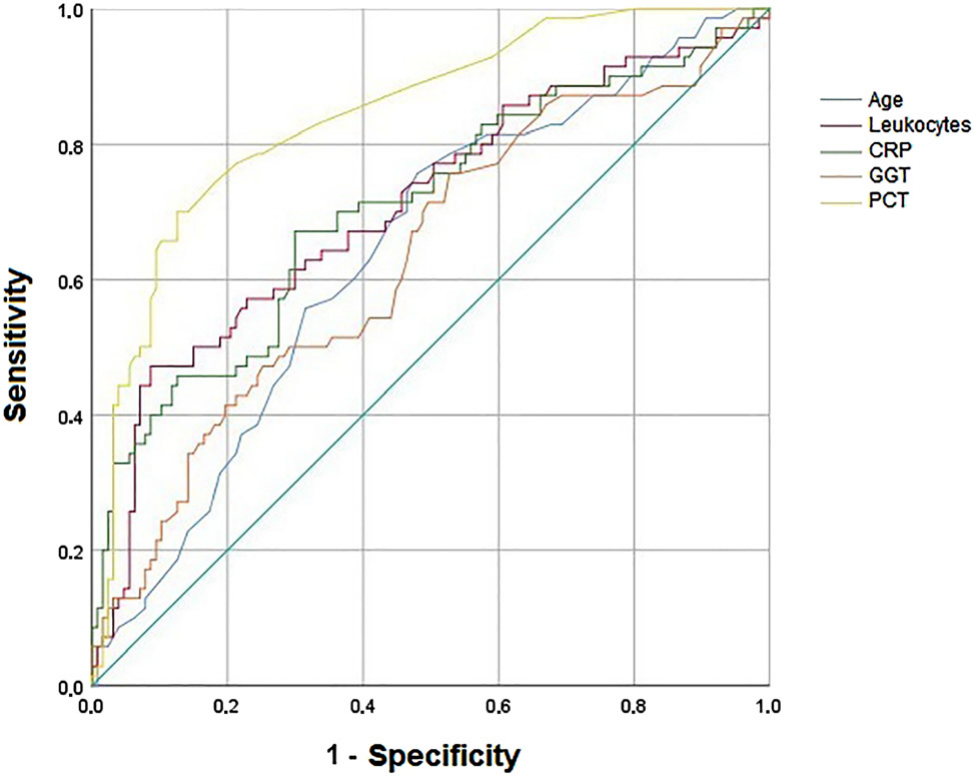
ROC curve of prognostic factors for severity. CRP, C-reactive protein; GGT, γ-glutamyltransferase; PCT, procalcitonin.

The ROC curve of the prognostic factors for severity included in the multivariate analysis is shown in Figure 1, whereas the AUC is displayed in Table 5.

**Table 5:** Area under the curve of prognostic factors for severity.

	AUC	95%CI	p-Value
Age	0.642	0.562–0.721	0.001
Leukocytes	0.708	0.630–0.787	<0.001
CRP	0.708	0.629–0.786	<0.001
GGT	0.632	0.550–0.714	0.002
PCT	0.844	0.787–0.901	<0.001

AUC, area under the curve; CI, confidence interval; CRP, C-reactive protein; GGT, γ-glutamyltransferase; PCT, procalcitonin.

The cut-off points with the highest Youden index obtained from the ROC curve were: age≥67 years (sensitivity 75.7% and specificity 52.0%), leukocytes≥9.44 × 10^9^/L (sensitivity 47.1% and specificity 91.3%), CRP≥90.5 mg/L (sensitivity 67.1% and specificity 70.1%), GGT≥28 U/L (sensitivity 75.7% and specificity 47.2%), and PCT≥0.19 ng/mL (sensitivity 70.0% and specificity 87.4%).

## Discussion

This study identifies prognostic factors for the progression of SARS-CoV-2 infection in hospitalized patients. More specifically, an advanced age (≥70 years), elevated levels of leukocytes (≥9.5 × 10^9^/L) and elevated concentrations of CRP (≥90 mg/L), GGT (≥30 U/L), and PCT (≥0.5 ng/mL) at admission were found to be associated with a higher risk for COVID-19 progression to severe disease.

Different mean or median ages of patients with SARS-CoV-2 infection are reported in previous studies, ranging from 47 to 62 years, and hypertension has been reported to be the most common comorbidity [9], [10]. In this study, the median age of inpatients with SARS-CoV-2 infection was 72 years, and more than half of the patients (58.4%) had hypertension (Table 1). More specifically, different studies identify advanced age as an important predictor of mortality in SARS-CoV and MERS-CoV. This study confirms that an advanced age is associated with a severe prognosis of SARS-CoV-2 infection [9], [10], [11].

The results of this study indicate that patients with a severe prognosis exhibited a higher leukocyte count and PCT concentrations. This is consistent with previous studies suggesting that the innate immune system of patients who end up dying is weakened due to a bacterial or viral condition secondary to SARS-CoV-2 infection, which causes an increase in blood cell count. Despite PCT synthesis is inhibited by interferon gamma, secreted in viral infections, elevated levels of this biomarker at admission indicate a higher risk of developing a bacterial infection [12], [13].

CRP is an acute phase reactant that is generally used as an inflammatory marker in clinical practice. It is synthesized in the liver under transcriptional control by interleukin-6, originated in the focus of inflammation. Data has been provided of elevated levels of CRP as a prognostic marker of severity of SARS-CoV-2 infection, as it is associated with progression of pulmonary infiltrates [9], [10], [11].

A variety of studies have been published revealing liver function alterations in patients with a severe SARS-CoV-2 infection [14], [15], [16]. This finding suggests that hepatocytes and cholangiocytes are potential targets of SARS-CoV-2 infection, as they express the ECA2 receptor, which is directly attacked by the virus. In addition, patients with previous liver dysfunction have been demonstrated to be more susceptible to this infection, because they have a high ECA2 expression, which requires close monitoring of liver function [14], [15]. Concentrations of biochemical indicators, including GGT, at admission are higher in critical patients, which is consistent with previous studies [15], [16].

Although CURB-65 is used to predict mortality in patients with CAP, most patients with pneumonia had 0–2 points on this scale (low/intermediate risk), including patients who were, admitted to the ICU, or died. Only 22.0% of critical patients had a CURB-65 of three points (high risk). This suggests that the CURB-65 scale may not be appropriate to predict COVID-19 severity [17].

The prognostic factors for the severity of COVID-19 described above have the advantage that they are routinely assayed in clinical practice and can be assessed in all hospitals, which ensures a rapid and easy provision of analytical results.

A limitation of this study is that high leukocyte, CRP, GGT, and PCT concentrations can be found in other inflammatory or infectious diseases. In addition, the relatively small sample size may result in bias. A large and multicentric study would be necessary to confirm the results obtained in this study.

In conclusion, an advanced age and elevated levels of leukocytes, CRP, GGT, and PCT at admission are predictors for the severity of SARS-CoV-2 infection and can be used to predict the course of COVID-19 at initial stages. This will help to optimize the management of patients according to their prognosis.
